# The recent progress of peptide regulators for the Wnt/β-catenin signaling pathway

**DOI:** 10.3389/fmed.2023.1164656

**Published:** 2023-06-16

**Authors:** Nan Zhang, Huaxing Shen, Baobao Chen, Honggang Hu, Chao Liu, Yan Chen, Wei Cong

**Affiliations:** ^1^School of Medicine or Institute of Translational Medicine, Shanghai University, Shanghai, China; ^2^Department of Pharmacy, Medical Supplies Center of People's Liberation Army (PLA) General Hospital, Beijing, China

**Keywords:** peptide regulators, small molecular drugs, biologics, Wnt/β-catenin signaling pathway, peptide regulators for Wnt/β-catenin, recent progress

## Abstract

Wnt signaling plays an important role in many biological processes such as stem cell self-renewal, cell proliferation, migration, and differentiation. The β-catenin-dependent signaling pathway mainly regulates cell proliferation, differentiation, and migration. In the Wnt/β-catenin signaling pathway, the Wnt family ligands transduce signals through LRP5/6 and Frizzled receptors to the Wnt/β-catenin signaling cascades. Wnt-targeted therapy has garnered extensive attention. The most commonly used approach in targeted therapy is small-molecule regulators. However, it is difficult for small-molecule regulators to make great progress due to their inherent defects. Therapeutic peptide regulators targeting the Wnt signaling pathway have become an alternative therapy, promising to fill the gaps in the clinical application of small-molecule regulators. In this review, we describe recent advances in peptide regulators for Wnt/β-catenin signaling.

## 1. Introduction

Wnt was initially discovered ~40 years ago (1), and the interest in Wnt signaling has increasingly risen. Wnt signaling plays an important role in many biological processes such as stem cell self-renewal, cell proliferation, migration, and differentiation (2). The Wnt signaling pathway is generally divided into canonical and non-canonical signaling pathways, i.e., β-catenin-dependent and β-catenin-independent signaling pathways, respectively (3). The β-catenin-dependent signaling pathway mainly regulates cell proliferation, differentiation, and migration (4). In the canonical signaling pathway, the Wnt family ligands transduce signals through LRP5/6 and Frizzled (FZD) receptors to the Wnt/β-catenin signaling cascades (5). The Porcupine (Porc) enzyme, a multiple-pass transmembrane *O*-acyltransferase in the endoplasmic reticulum, lipid-modifies the Wnt proteins. Through the Evi/Wntless multiple-pass transmembrane protein, the Wnt proteins are transported and secreted (6). The secreted Wnt ligands that are generally considered to stimulate the Wnt/β-catenin signaling pathway include Wnt1, Wnt2, Wnt3, Wnt8, and Wnt10 (7). Moreover, Wnt signals play a role by binding to the receptors during acute disease (8). The Wnt proteins commonly bind to the FZD receptor family, which are seven-transmembrane receptors, including 10 members. FZD has an intracellular C-terminal domain transducing downstream signals and an N-terminal cysteine-rich domain binding to Wnt ligands (7). In addition, LRP5/6 are co-receptors for FZD. LRP5/6 receptors lead to FZD receptor phosphorylation by forming a complex with the FZD receptor and Wnt proteins (9). R-spondin, a kind of Wnt agonist, potently enhances Wnt/β-catenin signals in *Xenopus* (10). After Wnt ligands bind to FZD/LRP receptors, the destruction complex residing in the cytoplasm regulates the stability of cytoplasmic β-catenin by binding and phosphorylating β-catenin (11). Axin, a type of tumor suppressor protein, acts as the scaffold of the destruction complex, interacting with the adenomatous polyposis coli (APC; a type of tumor suppressor protein) and casein kinase 1 (CK1) and glycogen synthase kinase 3 (GSK-3) (two types of the serine–threonine complex) (12). The cytoplasmic part of FZD interacts with Disheveled, a critical signal transducer in the Wnt/β-catenin signaling pathway, to promote interaction between Axin and LRP (13). The phosphorylated β-catenin then leaves the complex to be ubiquitinated by β-TrCP and then degraded by the proteasome. Subsequently, the destruction complex falls apart, and the β-catenin is stabilized and stimulates nuclear translocation (14). Upon Wnt signaling, β-catenin replaces Groucho from TCF/LEF and recruits histone and transcriptional coactivators for driving target gene expression (15).

Tissue homeostasis was controlled by the coordination of reprogramming, regeneration, and self-renewal of stem cells, in which the Wnt/β-catenin signaling pathway was tightly controlled in spatiotemporal patterns, and disruption of the pathway was found in many types of diseases (16): (1) Chronic obstructive pulmonary disease: a previous study indicated that the Wnt/β-catenin signaling pathway was involved in lung development, epithelial injury, and the repair process (17); (2) Neurodegenerative diseases: the Wnt signaling pathway was activated to enhance the formation, stabilization, and recycling of synapses and helped promote neurogenesis (18); (3) Bone disease: inactivation of the Wnt/β-catenin signaling pathway was inhibited to inactivate osteoblasts and bone resorption (19); (4) Pathological wound healing: activation of the Wnt/β-catenin signaling pathway enhanced wound closure by regulating follicle regeneration, differentiation and migration of keratinocytes, and proliferation of epidermal stem cells (20); and (5) Cancer: the Wnt/β-catenin signaling pathway plays an important role in maintaining stem cells in different tissues. Therefore, changes in its components may induce cancer such as colorectal cancer (21) and lung adenocarcinoma (22).

**Figure 1:**
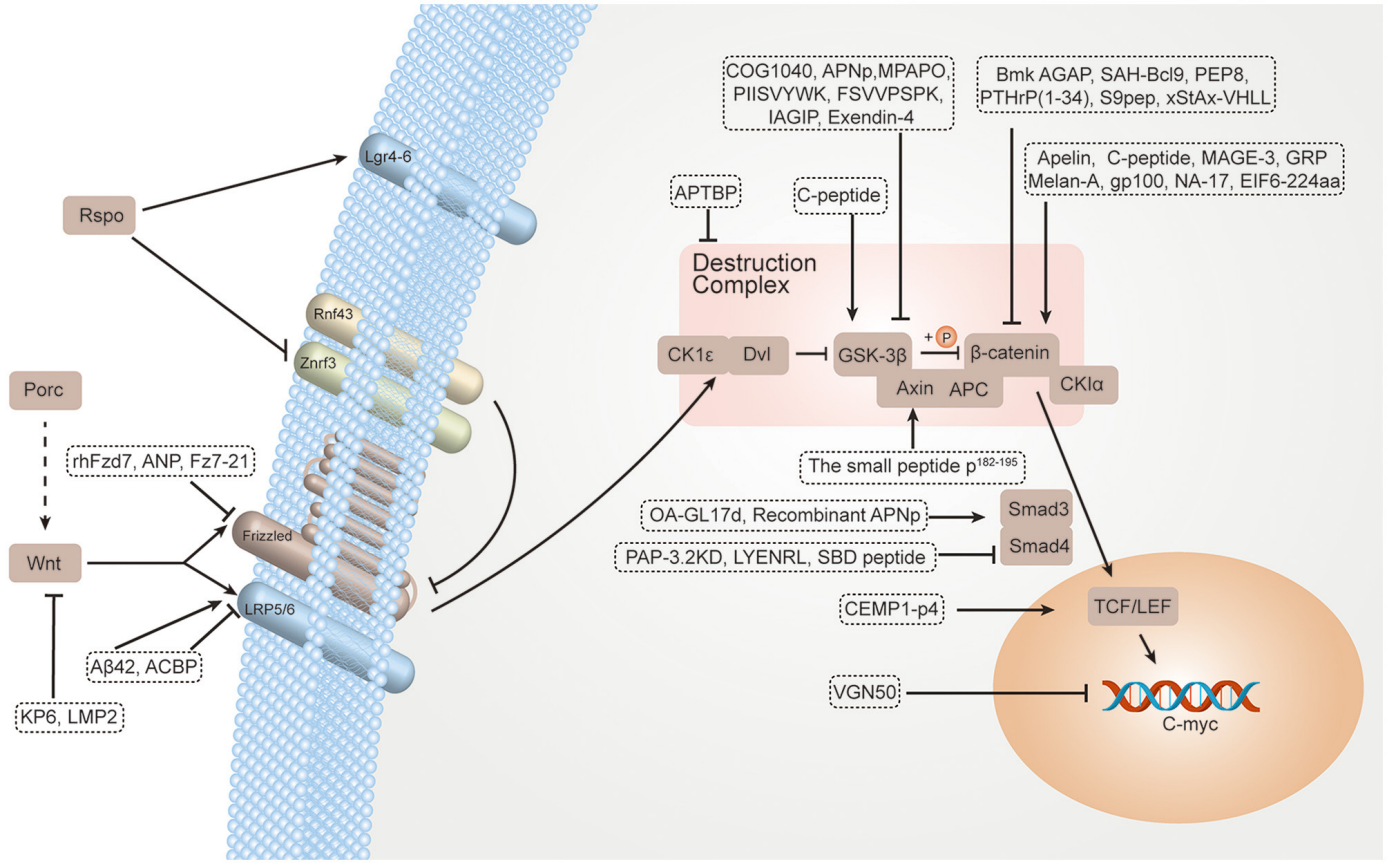
TPpeptide regulators for the Wnt/β-catenin signaling pathway.

Small-molecule regulators are commonly used in targeted therapy. IWP and SB-216763 can exert effects on upstream and downstream components for inhibiting and activating the Wnt/β-catenin signaling pathway (23). The compound IWP may inhibit Porc with high selectivity, especially inhibiting Wnt secretion (24). SB-216763 can inhibit the GSK-3 enzyme and promote Wnt target gene expression (25). In addition, biologics are also commonly used in targeted therapy. Advancements have been made in the development of biologics such as vantictumab (human anti-FZD antibody) (26) and ipafricept (a recombinant fusion protein that can compete for binding to Wnt ligands) (27). However, although several Wnt signaling pathway drugs have been used in clinical trials, no apparent advancement has been made. Most small molecular drugs interact with known, unintended, biological targets. These off-target interactions may lead to both preclinical and clinical toxic events (28). Moreover, biologics are expensive to prepare and have difficulty entering cells, and can only act on a few types of targets (29). Interestingly, many of the properties of peptide drugs are intermediate between those of small molecular drugs and biologics. Peptide drugs have many advantages such as high specificity, good efficacy, good safety, low immunogenicity, membrane permeability, and low cost. They are widely used in clinical practice and have broad prospects (30, 31). Therefore, therapeutic peptide regulators targeting the Wnt signaling pathway have become an alternative therapy, promising to fill the gaps left by small-molecule drugs and biologics. In this review, we describe recent advances in peptide regulators for the Wnt/β-catenin signaling pathway, aiming to use them better for the clinical treatment of diseases (Figure 1; Table 1).

**Table 1:** Therapeutics peptide regulators targeting the Wnt/β-catenin pathway components.

**Peptide**	**Target and associated human disease(s)**	**Key references**
KP6	Wnt Diabetic Kidney Disease	(32)
LMP2	Wnt Ischemia/Hypoxia-Induced BBB Injury	(33)
Aβ42	LRP5/6 Metabolic bone diseases	(34)
ACBP	LRP5/6 Colorectal Cancer	(35)
ANP	Frizzled TNBC	(36)
rhFzd7	Frizzled Tumors such as hepatoma	(37)
Fz7-21	Frizzled Cancer	(38)
The short peptide P^182−195^	Axin Cancer	(39)
COG1410	GSK-3β Early brain injury	(40)
APNp	GSK-3β Cerebral Ischemia-Reperfusion Injury	(41)
MPAPO	GSK-3β Corneal Wound	(42)
PIISVYWK	GSK-3β Bone-related Diseases	(43)
FSVVPSPK	GSK-3β Bone-related Diseases	(43)
IAGIP	GSK-3β Parkinson's Disease	(44)
Exendin-4	GSK-3β rtPA-induced hemorrhagic transformation after ischemic stroke	(45)
C-peptide	GSK-3β Decidualization in Human Endometrial Stromal Cells	(46)
APTBP	Destruction complex Hypoxia-Induced Cardiomyocyte Injury	(47)
BmK AGAP	β-catenin Breast Cancer	(48)
SAH-Bcl9_B_	β-catenin Osteoarthritis	(49)
xStAx-VHLL	β-catenin Intestinal Cancer	(50)
PTHrP (1-34)	β-catenin Bone resorption and osteogenesis of Dental follicle cells	(51)
S9pep	β-catenin Colon Cancer	(52)
PEP8	β-catenin Pancreatic Cancer	(53)
Apelin	β-catenin Fracture	(54)
EIF6-224a	β-catenin TNBC	(55)
GRP	β-catenin Fibrotic lung diseases	(56)
C-peptide	β-catenin Infertility	(57)
MAGE-3	β-catenin Melanoma	(58)
Melan-A	β-catenin Melanoma	(58)
gp100	β-catenin Melanoma	(58)
NA-17	β-catenin Melanoma	(58)
OA-GL17d	SMAD3 Skin Wound	(59)
APNp	SMAD3 Diabetes	(60)
PAP-3.2KD	SMAD4 Adriamycin-Induced Myocardial Injury	(61)
LYENRL	SMAD4 Hypertrophic scar	(62)
SBD peptide	SMAD4 Osteoporosis, arthritis, and periodontal diseases	(63)
CEMP1-p4	TCF/LEF Bone related diseases	(64)
VGN50	C-myc Leukemia and Lymphoma	(65)

## 2. Peptide regulators for Wnt ligands

Wnt signaling is important to many physiological, developmental, and disease processes (66). The Wnt signaling pathway is initiated by Wnt ligands. Wnt proteins are secreted proteins. They are synthesized and then lipid-modified by the Porc enzyme. Wnt proteins are further transported and secreted via the Evi/Wntless multiple-pass transmembrane (66, 67). There are 19 Wnt genes, which are divided into 20 conserved Wnt subfamilies (68). Wnt1, Wnt2, Wnt3, Wnt8, and Wnt19 ligands are frequently considered to activate the canonical (also named Wnt/β-catenin) signaling pathway (69, 70). Wnt4, Wnt5a, Wnt7, and Wnt11 are commonly considered to stimulate the non-canonical signaling pathway (71). In the absence of Wnt, β-catenin is not stabilized, and the Wnt/β-catenin signaling pathway is not activated. Therefore, the peptide regulators can inhibit the Wnt/β-catenin signaling pathway by inhibiting or binding to Wnt ligands (11).

The Klotho protein is an anti-aging protein abundantly expressed in normal kidneys. Klotho, a single-pass transmembrane protein, includes a short cytoplasmic tail (KL2) and a transmembrane segment (KL1). Based on the KL1 domain sequence of human Klotho, KP6 (sequence: QPVVTLYHWDLPQRLQDAYGGWANRALADH) was designed and synthesized by Chen et al. KP6 is 30 amino acids in length and contains a Wnt-binding motif ranging from Gln186 to His215 within the KL1 domain. KP6 can mimic Klotho's function. KP6 blocks the Wnt/β-catenin signaling pathway by binding to Wnt, which sequesters its ability to interact with LRP6. Furthermore, KP6 promotes survival and ameliorates kidney injury in diabetic mice (32).

**Figure 2:**
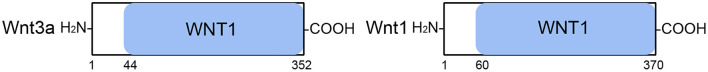
Schematic representation of Wnt protein.

A study indicated that the expression level of Wnt3a is high in myocardial hypertrophy mice and is positively related to cardiomyocyte apoptosis (72). The low-molecular-mass peptide 2 (LMP2, sequence: TYGPVFMCL) is a major catalytic subunit of the immunoproteasome. Dysregulation of the immunoproteasome has been linked with a variety of diseases (73). LMP2 can activate the Wnt/β-catenin signaling pathway by improving the expression levels of Wnt3a and β-catenin. The expression of tight junction proteins (occludin, claudin-1, and ZO-1) is downregulated to decrease blood–brain barrier (BBB) permeability after activating the pathway (33) (Figure 2; Table 2).

**Table 2:** Peptide regulators targeting Wnt.

**Name**	**Biological effect**	**Sequence**	**Function mechanism**
KP6	Inhibitor	QPVVTLYHWDLPQRL QDAYGGWANRALADH	Blocking the Wnt/β-catenin signaling pathway by binding to Wnts
LMP2	Inhibitor	TYGPVFMCL	Improving the expression level of wnt-3a and β-catenin

## 3. Peptide regulators for Frizzled/LRP receptors

When binding to target cells, Wnt proteins bind a receptor complex harboring FZD and LRP5/6 proteins (74). FZDs are a subset of seven-transmembrane proteins. They are the principal receptors of the Wnt/β-catenin signaling pathway (75). The *N*-terminal CRD domain of FZDs binds to Wnt ligands and LRP5/6 co-receptors. The C-terminus of FZDs is located in the cytoplasm, where it recruits and binds to Dsh for triggering subsequent signal cascades (76, 77). A previous study indicated that the knockdown of FZD7 decreases cell proliferation, invasion, and viability (78). FZD7 plays an important role in cell development, progression, and stem cell biology (79). Several approaches, such as adenoviral therapy (80) and monoclonal antibodies (81), have been applied to inhibit the Wnt/β-catenin pathway by targeting FZD.

### 3.1. Peptide regulators for Frizzled receptors

The recombinant extracellular peptide fragment (rhFzd7), including the CRD sequence, was synthesized from *Escherichia coli*. rhFzd7 may antagonize Fzd7 by competitively binding with Wnt ligands to inhibit Wnt/β-catenin signaling. Interestingly, rhFzd7 exhibited a high affinity with Wnt3a (K_d_ = 3.41 × 10^−8^ mM). Moreover, rhFzd7 may effectively repress angiogenesis, proliferation, and invasion and induce apoptosis of triple-negative breast cancer cells (37).

Ligands from a linear peptide library that bound to the Fc-tagged hFzd7 CRD were identified. Subsequently, five peptides were synthesized. Among them, Fz7-21 (sequence: LPSDDLEFWCHVMY) was the most potent peptide. A 5-carboxyfluorescein-labeled version of Fz7-21 (5FMA-Fz7-21) exhibited subtype-selective binding to Fc-tagged FZD1, FZD2, and FZD7 CRDs with half-maximal effective concentration values (19-58 nM). dFz7-21 exhibited an ~40-fold improvement in inhibiting Wnt3a signaling in HER293 cells as compared with monomeric Fz7-21. The crystal structure indicated that the dimeric form of Fz7-21 (dfz7-21) exhibits improved activity. dFz7-21 exhibited tight binding to the hFzd7 CRD (K_d_ = 3 nM). The dFz7-21 may inhibit Wnt signaling by perturbing the formation of the Wnt–FZD–LRP ternary complex to further impair stem cell function (38).

**Figure 3:**
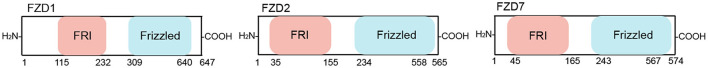
Schematic representation of Frizzled protein. The Frizzled possesses two functional domains, including Frizzled (FRI) and Frizzled/Smoothened family membrane region (Frizzled).

Atrial natriuretic peptide (ANP, sequence: SLRRSSCFGGRMDRIGAQSGL GCNSFRY) was synthesized as an inactive precursor (pro-ANP) that was converted to a mature active peptide after proteolytic cleavage by the membrane-related serine protease Corin, in which the extracellular region included Fzd1 and Fzd2 (receptors for Wnt signaling) (82). ANP may induce β-catenin stabilization and nuclear translation possibly by direct interaction with the Frizzled receptors. The Wnt/β-catenin signaling cascade was activated, thereby exerting a neuroprotective effect in cellular systems mimicking the neurodegeneration in Parkinson's disease (36) (Figure 3; Table 3).

**Table 3:** Peptide regulators targeting frizzled.

**Name**	**Biological effect**	**Sequence**	**Function mechanism**
ANP	Activator	SLRRSSCFGGRMD RIGAQSGLGCNSFRY	Inducing β-catenin stabilization and nuclear translation via interacting with the Frizzled
rhFzd7	Activator		Antagonizing Fzd7 by competitively binding with Wnt ligand
Fz7-21	Activator	LPSDDLEFWCHVMY	Perturbing the formation of the Wnt-FZD-LRP ternary complex

### 3.2. Peptide regulators for LRP receptor

ACBP is a polypeptide that was identified from goat spleen extract after immunization with gastric cancer lysates (83). ACBP can inhibit phosphor-LRP6 and stimulate β-catenin, further leading to active β-catenin in the cytoplasm. Subsequently, the Wnt/β-catenin signaling pathway was inhibited to suppress the invasion, migration, and proliferation of colorectal cancer (35).

**Figure 4:**
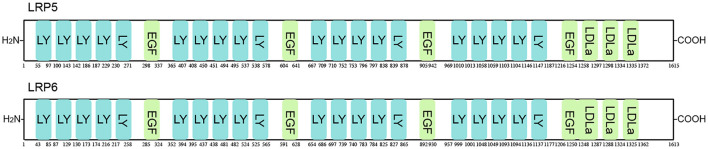
Schematic representation of LRP5/6 protein. LRP5/6 possesses 27 functional domains, including low-density lipoprotein receptor YWTD domain (LY), epidermal growth factor-like domain (EGF), and low-density lipoprotein receptor domain class A (LDLa).

Amyloid β peptide (Aβ) is a small proteolytic fragment (40 to 42 amino acids) consisting of a glycosylated transmembrane protein and amyloid precursor protein (84). Aβ42 (sequence: DAEFGHDSGFEVRHQKLVFFAEDVGSNKGAIIGLMVGGVVIA) can bind to LRP5/6 and consequently upregulate the Wnt/β-catenin signaling pathway. TCF1, the downstream target of Wnt signaling, is activated to further enhance the expression of matrix mineralization and osteogenic markers (Runx2 and osteocalcin) (34) (Figure 4; Table 4).

**Table 4:** Peptide regulators targeting LRP5/6.

**Name**	**Biological effect**	**Sequence**	**Function mechanism**
ACBP	Activator		Inhibiting phosphor-LRP6 and further upregulating active β-catenin
Aβ42	Activator	DAEFGHDSGFEVRHQKLVFFAEDVGSNKGAIIGLMVGGVVIA	Binding to LRP5/6 and further activating TCF1

## 4. Peptide regulators for the cytoplasmic Axin2/GSK-3β destruction complex

The destruction complex may be a dynamic multiprotein assembly. Its core components include the serine-threonine kinase GSK-3, APC protein, scaffolding protein Axin, CK1, and E3-ubiquitin ligase β-TrCP (11). Peptide regulators may control the production of stable β-catenin by regulating whether the destruction complex falls apart.

### 4.1. Peptide regulators for Axin

Axin is a largely unstructured, flexible protein that contains β-catenin, GSK-3, and CK1-binding sites. Axin is a key control point for catenin destruction because of its ability to promote the phosphorylation of β-catenin (12). A previous study indicated that Axin mutations lead to inappropriate β-catenin–mediated transcription (85). In addition, overexpression of Axin in colon cancer cells can reduce the accumulation of β-catenin (86).

**Figure 5:**
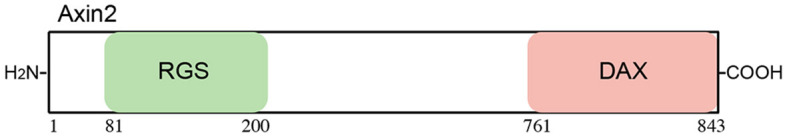
Schematic representation of Axin2 protein. Axin2 possesses two functional domains, including regulators of the G protein signaling domain (RGS) and the domain present in Disheveled and Axin (DAX).

Conductin, also named Axin2, is an Axin paralog exhibiting similar domain architecture (39). In contrast to Axin, there are no indications for Axin2 polymerization. Axin2 appears to be diffusely distributed in the cytoplasm. In the lower polymerization of Axin2, it is less active in degrading β-catenin than Axin (87). The regulators of the G protein signaling (RGS) domain of Axin2 may prevent Axin2 polymerization, because of the considerable molecular structure of the RGS domain. A short peptide P^182−195^ (sequence: MEENAYQVFLTSDI) is designed and synthesized, which is centered on aggregation preventing polymerization and consists of Axin amino acids^182−185^. The short peptide can induce the polymerization of Axin and further block Wnt signaling and colorectal cancer growth (39) (Figure 5; Tables 5, 6).

**Table 5:** Peptide regulator targeting Axin2.

**Name**	**Biological effect**	**Sequence**	**Function mechanism**
The short peptide P^182−195^	Activator	MEENAYQVFLTSDI	Inducing polymerization of axin and further blocking Wnt signaling

**Table 6:** Peptide regulator targeting the destruction complex.

**Name**	**Biological effect**	**Sequence**	**Function mechanism**
APTBP	Activator	VKAGFAWTANQQLS	Disrupting the formation of the destruction complex

### 4.2. Peptide regulators for GSK-3β

GSK-3β is an evolutionarily conserved serine/threonine kinase functioning in numerous cellular processes such as cell cycle, DNA repair, cell proliferation, and metabolic and signaling pathways (88). GSK-3β can phosphorylate the serine of the PPPSP motif found in many Wnt signaling components (86). When its function is inhibited, the Wnt/β-catenin signaling pathway can be activated (86). Peptides can regulate Wnt/β-catenin by activating or inhibiting GSK-3β.

#### 4.2.1. Activators

The C-peptide (sequence: EVEDPQVPQLELGGGPEAGDLQTLALEVARQ) was initially identified in 1970 (89). It is cleaved from proinsulin during proteolytic processing. It can activate protein–protein interaction protein phosphatase 1 activity using a GSK-3β-dependent mechanism, which dephosphorylates GSK-3β at Ser9. The cross-activation between GSK-3β and protein phosphatase 1 decreases apoptosis and increases cellular senescence, leading to the inhibition of decidualization (46).

#### 4.2.2. Inhibitors

Exendin-4 (EX-4, sequence: HGEGTFTSDLSKQMEEEAVRLFIEWLKNGGPSSGAPPPS) is an agonist of the glucagon-like peptide-1 receptor. EX-4 can reduce the degradation of β-catenin by inhibiting the expression of GSK-3β. The Wnt/β-catenin signaling pathway was activated to further protect the integrity of the BBB (45).

Three antioxidant peptides, namely, PIIVYWK (1004.57 Da, P1), TTANIEDRR (1074.54 Da, P2), and FSVVPSPK (860.09 Da, P3), were identified from a peptide fraction of 1–5 kDa obtained from blue mussels by peptic hydrolysis. It was found that the P1, P2, and P3 antioxidant peptides had a higher 2,2-diphenyl-1-picrylhydrazyl (DPPH) radical scavenging activity than previously reported antioxidant peptides from blue mussels (90, 91). In addition, the IC_50_ value against DPPH of P1 and P3 (0.71 ± 0.01 and 1.09 ± 0.01 mM, respectively) was higher than that of the P2 antioxidant peptide (2.33 ± 0.56 mM). Therefore, P1 and P3 antioxidant peptides warrant further study (92). P1 and P3 antioxidant peptides may enhance protein expression of Wnt1 and Wnt3a and downregulate protein expression of GSK-3β, ultimately stimulating the nuclear translocation of β-catenin. Subsequently, the Wnt/β-catenin signaling pathway was activated to promote human bone marrow-derived mesenchymal stem cell differentiation into osteoblasts (43).

Pituitary adenylate cyclase-activating polypeptide (PACAP) is a type of peptide hormone superfamily, including glucagon-like peptides, glucagon, and vasoactive intestinal peptides (93), which exists in the form of PACAP38 and PACAP27 *in vivo* (94). PACAP has poor stability *in vivo*, and its half-life is less than 10 min. PACAP27-derived mutant peptide (named MPAPO, MHSDGIFTDSYSRYRKQLAVKKYLA AVKK) (95) was designed and synthesized. MPAPO, compared with PACAP27, has the following improvements: first, the binding and specific agonistic effects of MPAPO to the PAC1 receptor are significantly enhanced. Second, MPAPO has a longer half-life, more stable activity, and better stability. MPAPO can phosphorylate GSK-3β, which, once phosphorylated, loses the ability to phosphorylate β-catenin. β-Catenin enters the nucleus and drives the transcription of target genes to accelerate cell cycle progression and promote cell proliferation (42).

IAGIP (sequence: EPVPPPPTPRSSRHDSGLDSMKD) was designed by linking the IKK-recognized (inflammation-responsive) motif to the LRP peptide (the GSK-3β inhibitory motif) (95). IAGIP can directly target the N-terminal region of GSK-3β, which includes Ser9, and can block GSK-3β activity. The inhibition of excessive GSK-3β activity provided a possible approach to controlling neuroinflammation in neurodegenerative diseases (44).

Adiponectin (APN) is a 247-amino-acid polypeptide with a molecular weight of 30 kDa. BBB permeability was restricted by APN under physiological conditions. The APN peptide (APNp, sequence: LQVYGDGDHNGLYADNVN) was chemically synthesized based on the functional area in the globular domain of APN (96). APNp could effectively cross the BBB and bind with the intercephalic APN receptors to reliably simulate and maintain the function of endogenous APN. APNp can upregulate the phosphorylation of GSK-3β to improve neurological function and exert antiapoptotic, anti-inflammatory, and antioxidative effects against cerebral ischemia-reperfusion injury (41).

p-GSK-3β can be upregulated by exogenous administration of apolipoprotein E (apoE), thereby promoting neuronal survival. However, apoE has difficulty crossing the BBB because of its 34-kDa molecular weight, thereby limiting its translational study (97). COG1410 (sequence: AS(Aib)LRKL(Aib)KRLL, Aib: 2-Aminoisobutyric acid), a modified apoE mimetic peptide, was designed with a composition of apoE residues 138–149 and the modification of two residues. COG1410 could effectively cross the BBB and have a long-lasting effect (98). COG1410 promoted neuronal autophagy by promoting the phosphorylation of GSK-3β (40).

**Figure 6:**
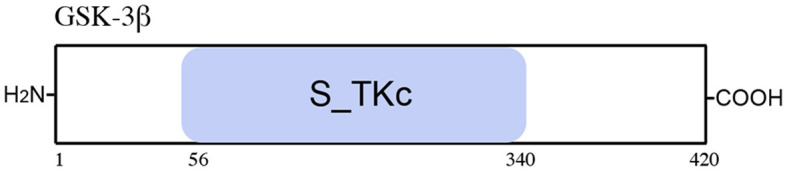
Schematic representation of the GSK-3β protein. The GSK-3β includes one functional domain, serine/threonine protein kinases, and a catalytic domain (S_TKc).

A novel peptide purified from the hydrolysates of tuna backbone protein (APTBP, VKAGFAWTANQQLS) was identified to have strong antioxidant activity (99). APTBP had high stability and a significant amphipathic characteristic. APTBP with a cell-penetrating peptide (CPP: GRKKRRQRRRPPQ) was chemically synthesized and derived from HIV-1 Tat (34–39, 80–86) attached to the N-terminus. The expression of APC and Axin2 was decreased by treatment with CPP-APTBP, which indicated that the CPP–APTBP function could be related to the disruption of the destruction complex. Subsequently, the activity of Wnt/β-catenin could be restored by CPP-APTBP. The accumulation of β-catenin can ameliorate hypoxia-induced cardiomyocyte apoptosis (47) (Figure 6; Table 7).

**Table 7:** Peptide regulators targeting GSK-3β.

**Name**	**Biological effect**	**Sequence**	**Function mechanism**
COG1410	Inhibitor	AS(Aib)LRKL(Aib)KRLL^a^	Promoting the phosphorylation of GSK-3β
APNp	Inhibitor	LQVYGDGDHNGLYADNVN	Upregulating the phosphorylation of GSK-3β
MPAPO	Inhibitor	MHSDGIFTDSYSRYRKQLAV KKYLAAVKK	Phosphorylating GSK3β
PIISVYWK	Inhibitor	PIIVYWK	Downregulating the protein expression of GSK3β
FSVVPSPK	Inhibitor	FSVVPSPK	Downregulating the protein expression of GSK3β
IAGIP	Inhibitor	EPVPPPPTPRSS RHDSGLDSMKD	Targeting the N-terminal region of GSK-3β to block GSK-3β activity
Exendin-4	Inhibitor	HGEGTFTSDLSKQMEEEAV RLFIEWLKNGGPSSGAPPPS	Inhibiting the expression of GSK-3β

^a^Aib: 2-Aminoisobutyric acid.

## 5. Peptide regulators for β-catenin and its target genes

β-Catenin is a type of plasma membrane-associated protein, and it plays a dual role in cellular signaling. On the one hand, β-catenin can stabilize cell–cell contact. On the other hand, it plays a role in activating gene transcription after β-catenin translocation to the nucleus (100). β-Catenin plays a crucial role in a multitude of homeostatic and developmental mechanisms and is the key nuclear effector of the Wnt/β-catenin signaling pathway (101). The stability of cytoplasmic β-catenin has a role in the signal output of the Wnt/β-catenin signaling pathway. Some peptide regulators can regulate the Wnt/β-catenin signaling pathway by activating or inhibiting β-catenin.

### 5.1. Peptide regulators for β-catenin

#### 5.1.1. Activators

Apelin is the endogenous ligand for APJ, which is a seven-transmembrane G protein-coupled receptor (102). Apelin consists of numerous isoforms, including Apelin-12, 13, 17, and 36, which are all derived from the C-terminal fragment of the Apelin pre-proprotein with 77 amino acids (103). Among them, Apelin-13 needs further study because it exhibits the most active isoforms binding to the APJ receptor (104). Apelin-13 (sequence: QRPRLSHKGPMPF) could activate the Wnt/β-catenin signaling cascade by increasing the expression levels of total β-catenin and active β-catenin, thereby promoting osteogenic differentiation of human bone marrow-derived mesenchymal stem cells (54).

A novel peptide, EIF6-224 amino acid (EIF6-224aa, sequence: PDAGREV AESSLGLR), was encoded by circ-EIF6. EIF6-224aa is responsible for the oncogenic effects of circ-EIF6. EIF6-224aa can interact with the oncogene MYH9 and subsequently decrease MYH9 degradation by upregulating the expression of β-catenin. The overexpression of MYH9 can enhance the metastasis of TNBC cells and promote the progression of TNBC (55).

Gastrin-releasing peptide is a neuropeptide that acts through G protein-coupled receptors, which stimulate the proliferation of mesenchymal cells in the fetal monkey lung (105). GRP increased active β-catenin and total β-catenin levels and β-catenin mRNA expression. Myofibroblast differentiation was promoted after the activation of the pathway (56).

The C-peptide (sequence: EVEDPQVPQLELGGGPEAGDLQTLALEVARQ) initially identified was cleaved from proinsulin during proteolytic processing in 1970 (89). C-peptide may activate the Wnt/β-catenin signaling pathway by inducing nuclear translocation of β-catenin, except targeting GSK-3β. β-Catenin in the nucleus directly regulates the transcription of MMP9/TIMP1/TIMP3, thereby promoting the migration of human endometrial stromal cells (UnD-ESCs) (57).

The peptide vaccine for melanoma includes four peptides: Melan-A (sequence: AAGI GILTV), gp100 (sequence: KTWGQYWQV), MAGE-3 (sequence: FLWGPRALV), and NA-17 (sequence: VLPDVFIRCV). Melan-A has a “bulged” and “stretched” conformation. To identify the MART-1 epitopes for tumor-infiltrating lymphocytes, 23 peptides were selected based on the known peptide-binding motif to HLA-A2.1 and synthesized (>90% purity). Among them, Malan-A could most effectively sensitize T2 cells for lysis. Gp100 is a melanocyte-derived lineage-restricted intracellular glycoprotein that can be presented on the cell surface in association with major histocompatibility complex molecules in T lymphocytes (106). Several peptides generated from gp100 have been isolated and characterized. Among them, gp154-162 (sequence: KTWGQYWQV) necessitated further study because it was the most abundant at the cell surface (107). Six genes of the MAGE family, namely, MAGE-1, 2, 3, 4, 6, and 12, were found to be expressed at a high level in many tumor types of various histological origins (108). A MAGE-3 antigen presented by HLA-A2, which is the most common HLA allele, might therefore be widely used in the immunotherapy of melanoma. Based on the initial screening for binding to HLA-A2, the peptide (MAGE-3, sequence: FLWGPRALV) was synthesized (109). NA17 was designed and synthesized based on *N*-acetylglucosaminyltransferase-V from the GnT-V gene intron, nt 38–67, which appeared to induce peptide-specific recognition of target cells (110). After the vaccine is injected into patients with metastatic melanoma, the β-catenin protein stabilization is enhanced. In addition, the high-level expression of β-catenin is found in recurrent tumors after developing new treatment-resistant metastases. Therefore, the peptides in the vaccine are considered to be closely related to β-catenin (58).

#### 5.1.2. Inhibitors

The first *Buthus martensii* Karsch (BmK) analgesic peptide was identified from a venom (111). Since then, more BmK analgesic peptides, including BmK AGAP, have been purified (112). BmK AGAP (sequence: VRDGYIADDKNCA YFCGRNAYCDDECEKNGAESGYCQWAGVYGNACWCYKLPDKVPIRVPGKCNGG) belongs to a group of long-chain scorpion peptides, and it has a molecular mass of 7,142 Da (113). The rBmK has a molecular scaffold consisting of an α-helix and three-stranded antiparallel β-sheets, resulting in a compact core and several loops and c-turns extending outside (114). It can target PTX3 and further decrease the expression of β-catenin to influence the Wnt/β-catenin signaling pathway. BmK AGAP can effectively inhibit breast cancer cells (IC_50_ for MCF-7 = 40 μM; IC_50_ for MDA-MB-231 cells = 50 μM). In addition, the expression of the target gene of β-catenin, E-cadherin, is upregulated, thereby reducing breast cancer cell stemness and epithelial–mesenchymal transition (48).

Verdine Lab designed and prepared xStAx, which is a type of stapled peptide, modeled after an Axin-derived peptide motif. Stapled peptides exhibited increased binding affinity, functional and conformational stability, and cell penetration via endocytosis. Based on design criteria, stapled Axin (StAx) CBD StAx-1, 2, and 3 were further designed. Among them, StAx-3 exhibited the greatest α-helicity (51%). Moreover, fStAx-3 exhibited significantly enhanced affinity for β-catenin (K_d_ = 60 ± 2 nM). The StAx-3 sequence was thus selected for further optimization. In a parallel series of experiments, affinity optimization of the Axin peptide sequence was examined. In addition, 32 new sequences were obtained. Among them, peptide fStAx-35R (sequence: RRWPRSILDSHVRRVWR) with high binding affinity for β-catenin (K_d_ = 53 ± 9 nM) and positive charge was obtained upon the addition of Arg and Trp at positions 467 and 468 (fStAx-34) and addition of arginine residues. Proteolysis-targeting chimera is a useful technology to degrade proteins *in vivo*. In this study, a novel proteolysis-targeting chimera β-catenin degrader was designed by coupling xStAx-35R with a von Hippel–Lindau protein (VHL) ligand via an Ahx chemical linker, termed xStAx-VHLL. xStAx-VHLL can sustain the degradation of β-catenin and significantly inhibit the Wnt/β-catenin signaling pathway (50).

PTHrP is synthesized and expressed by various cells and tissues. After posttranslational processing, PTPrp could generate a variety of fragments, including PTHrP (1-34, sequence: AVSEHQLLHDKGKSIQDLRRRFFLHHLIAEIHTA), PTHrP (38-94), and PTHrP (107–139) peptides. Among them, PTHrP (1–34) can bind to the PTHrP receptor (PTH1R), a common G protein-coupled receptor. PTHrP (1–34) warrants further study because of its important role in tooth and bone development (115). PTHrP (1–34) can inactivate the Wnt/β-catenin pathway and further inhibit the expression of osteogenic-related genes (ALP, RUNX2, BSP, and OPN) in dental follicle cells. Ultimately, osteogenesis of dental follicle cells is inhibited (51). SOX9, the most extensively studied SOX family member, was both a downstream target and an inhibitor of the Wnt/β-catenin signaling pathway (116).

S9pep (sequence: ADSPHSSSGMSEVHSPGEHSG) was designed and synthesized based on the amino acid sequence located in the SOX9 central region. The peptide has S9pep, which could inhibit the expression of c-Myc (its oncogenic target gene) by interacting with and relocating β-catenin from the chromatin to the cytosol, like SOX9 (52).

A deca-peptide library of the DXMXXPQQTE sequence with 6,859 members was produced in MATLAB, where X denotes one of the 19 standard amino acids, except for cysteine. PEP8 (sequence: DEMEEPQQTE) was selected and synthesized after a series of screenings, for example, the calculation of affinity energy values for each amino acid and the root mean square deviation from the reference conformation. Peptide PEP8 expanded the size of the repository of druggable target proteins. PEP8 can inhibit the formation of the β-catenin/LRH-1 complex, thereby inhibiting the Wnt/β-catenin signaling pathway. In addition, PEP8 can inhibit the growth of AsPC-1 cells (IC_50_ value = 288 μM). To promote entry into the cells, TAT-PEP8 (sequence: GRKKRRQRRRPPQDEMEEPQQTE) was designed and synthesized by attaching the cell-penetrating peptide TAT to PEP8. TAT-PEP8 can inhibit the growth of AsPC-1 cells (IC_50_ value = 111 μM) (53).

**Figure 7:**
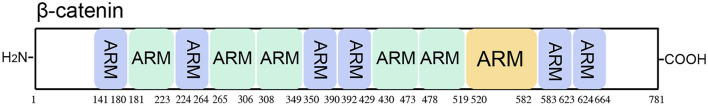
Schematic representation of the β-catenin protein. β-catenin includes 12 functional domains consisting of Armadillo/β-catenin-like repeats (ARM).

B-cell lymphoma 9 (BCL9) is a type of co-activator of β-catenin, which was first discovered in some B-cell malignant tumors (117). Peptide regulators targeted in the β-catenin/BCL9 interaction have garnered extensive attention, such as SAH-BCL9. Hydrocarbon stapling was applied to generate cell-permeable α-helical peptides of the BCL9 HD2 domain for *in vivo* and *in vitro* studies (118, 119). Non-natural amino acids with olefinic side chains were substituted at (*i, i*+4) positions, followed by ruthenium-catalyzed olefin metathesis to yield SAH-BCL9 peptides A through C. Among SAH-BCL9_A−*C*_, SAH-BCL9_B_ (sequence: LSQEQLEHRERSLXTRXIQRMLF) was identified as the most effective β-catenin-targeting peptide. In addition, circular dichroism analysis confirmed that hydrocarbon stapling consistently enhanced peptide α-helicity compared with the corresponding unmodified peptide (BCL9_HD2_). SAH-BCL9 could target β-catenin in cells with a high affinity (IC_50_ = 135 nM) and selectively disrupt the BCL9/β-catenin complex in a dose-dependent manner. Blockade of these interactions inhibited β-catenin-dependent transcriptional activity and target gene expression and suppressed tumor cell growth, metastasis, and angiogenesis (49) (Figure 7; Table 8).

**Table 8:** Peptide regulators targeting β-catenin.

**Name**	**Biological effect**	**Sequence**	**Function mechanism**
BmK AGAP	Inhibitor	VRDGYIADDKNCAYFCGRNAYCDDECEKNG AESGYCQWAGVYGNACWCYKLPDKVPIRVPGKCNGG	Targeting PTX3 and further decreasing the expression of β-catenin. IC_50_ for MCF-7 cells: 40 μM; IC_50_ for MDA-MB-231 cells: 50 μM; demonstrated by MTT assay
SAH-Bcl9_B_	Inhibitor	LSQEQLEHRERSLS_5_TLRS_5_IQRMLF^a^	Targeting β-catenin and selectively disrupting the BCL9/β-catenin complex. IC_50_ =135 nM (for Colo 320 and MM1S cells, demonstrated by GST-pull-down assay)
xStAx-VHLL	Inhibitor	RRWPRS_5_ILDS_5_HVRRVWR-Ahx-ALAPYIP^b^	Sustaining degradation of β-catenin. IC_50_ =135 nM (for Colo 320 and MM1S cells, demonstrated by GST-pull-down assay)
PTHrP(1-34)	Inhibitor	AVSEHQLLHDKGKSIQDLRRRFFLHHLIAEIHTA	Inactivating the Wnt/β-catenin pathway
S9pep	Inhibitor	ADSPHSSSGMSEVHSPGEHSG	Interacting with and relocating β-catenin
PEP8	Inhibitor	DEMEEPQQTE	Inhibiting the formation of the β-catenin/LRH-1 complex. IC_50_ =288 μM (for AsPC-1 cells, demonstrated by MTT assay)
Apelin	Activator	QRPRLSHKGPMPF	Increasing the expression levels of total β-catenin and active β-catenin
EIF6-224a	Activator	PDAGREVAESSLGLR	Upregulating the expression of β-catenin
GRP	Activator	VPLPAGGGTVLTKMYPRGNHWAVGHLM	Increasing active β-catenin, total β-catenin levels, and β-catenin mRNA expression
C-peptide	Activator	EVEDPQVPQLELGGGPEAGDLQTLALEVARQ	Inducing nuclear translocation of β-catenin
MAGE-3	Activator	FLWGPRALV	Enhancing β-catenin protein stabilization
Melan-A	Activator	AAGIGILTV	Enhancing β-catenin protein stabilization
gp100	Activator	KTWGQYWQV	Enhancing β-catenin protein stabilization
NA-17	Activator	VLPDVFIRCV	Enhancing β-catenin protein stabilization

^a^S_5_: (S)-α-(4-pentenyl) alanine, a pair of S_5_ was coupled through the ring-closing metathesis reaction. ^b^Ahx: 6-aminocaproic acid; S_5_: (S)-α-(4-pentenyl) alanine, a pair of S_5_ was coupled through the ring-closing metathesis reaction.

### 5.2. Peptide regulators for c-Myc

**Figure 8:**
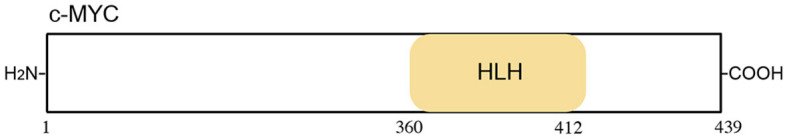
Schematic representation of the c-Myc protein. The c-Myc includes 12 functional domains, Helix loop helix domain (HLH).

c-Myc (a type of target gene of β-catenin) is a type of proto-oncogene. It encodes a phosphoprotein that acts in cellular transformation, apoptosis, and cell cycle progression (120). When Kaposi sarcoma herpesvirus (KSHV) reactivation begins, RNA polymerase II molecules are effectively recruited to viral episomes and form a complex with a viral protein, KSHV replication, and transactivator (K-Rta), for vital gene expression (121). Moreover, KSHV reactivation induced the interaction of coactivators with both RNA polymerase II and K-Rta. A small peptide was designed based on the binding interface of K-Rta and the cellular coactivator complex. The homologous protein sequences were extracted from other gamma-herpesvirus homologs and a bacterial transcription factor. Based on the conserved protein sequence, a series of K-Rta peptides (P1-P5) were synthesized. Among them, P1 (renamed VGN50, sequence: LSSILQGLYQLDT) needs further study because of its major effect on cell viability and viral replication. VGN50 could recruit the SWI/SNF complex by engaging the MYC promoter, resulting in the downregulation of both MYC and MYC-target gene transcription. Ultimately, cell proliferation in leukemia and lymphoma is inhibited (65) (Figure 8; Table 9).

**Table 9:** Peptide regulator targeting c-Myc.

**Name**	**Biological effect**	**Sequence**	**Function mechanism**
VGN50	Inhibitor	LSSILQGLYQLDT	Recruiting the SWI/SNF complex by engaging the MYC promoter and downregulating MYC

## 6. Peptide regulators for TCF/LEF

Although it is challenging to directly target β-catenin, it is possible to indirectly activate it through transcriptional cofactors and by controlling its stabilization. TCF and LEF are transcription factors mainly involved in the Wnt/β-catenin signaling pathway (122). The mammalian TCF/LEF consists of four nuclear factors, namely, TCF7L1, TCF7L2, LEF1, and TCF7 (also known as TCF3, TCF4, LEF1, and TCF1, respectively) (123). The activation of target genes by the β-catenin/TCF complex has been considered the main mode of the Wnt/β-catenin signaling pathway (124). LEF1 is widely expressed during mouse embryonic development. Moreover, its expression is tissue-specific in adults (125). A full-length LEF isoform that interacts with β-catenin is regulated by β-catenin-TCF complexes (122).

**Figure 9:**
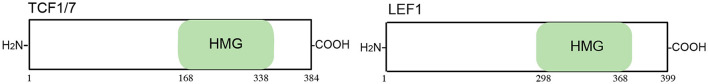
Schematic representation of the TCF/LEF protein. TCF/LEF includes 12 functional domains, a high mobility group (HMG).

Cementum markers, such as cementum protein 1 (CEMP1), were shown to promote the proliferation and differentiation of periodontal ligament cells toward a “mineralizing-like” phenotype (126). The secondary structure analysis of CEMP1 demonstrated that it had a random coil structure. A short peptide (named CEMP1-p4, sequence: QGQGDTEDGRMTLMG) was designed and synthesized based on the C-terminus of CEMP1, activating β-catenin signaling in human oral mucosa stem cells. CEMP1-p4 also had a random coil structure and could mimic the biological capabilities of CEMP1. CEMP1-p4 may activate the Wnt/β-catenin pathway by enhancing the expression of β-catenin, decreasing the expression of GSK-3β, and activating downstream transcriptional factors (TCF1/7 and LEF1). Subsequently, the expression of mineralization-related markers (OSX, RUNX2, IBSP, and OCN) was upregulated in human oral mucosal stem cells at both the mRNA and protein levels, driving the differentiation of HOMSCs to a mineralizing-like phenotype (64) (Figure 9; Table 10).

**Table 10:** Peptide regulator targeting TCF/LEF.

**Name**	**Biological effect**	**Sequence**	**Function mechanism**
CEMP1-p4	Activator	QGQGDTEDGR MTLMG	Activating downstream transcriptional factors

## 7. Peptide regulators for SMAD3/4

There is a crosstalk between the TGF-β/SMAD signaling pathway and the Wnt/β-catenin signaling pathway (127). TGF-β in the background of elevated SMAD3 enhances canonical Wnt/β-catenin signaling (128). The impact of increased β-catenin mRNA associated with SMAD4 loss is biologically significant (129).

OA-GL17d (sequence: GLFKWHPRCGEEQSMWT), a new natural peptide homodimer, was identified from the skin secretions of *Oreochromis andersonii*. OA-GL17d was degraded after 10 h, and its half-life was ~1.86 h, much longer than that of some peptides (130). It may act on TGF-β1 through miR-663a to activate the SMAD signaling pathway, thereby promoting skin wound regeneration (59).

The pilose antler polypeptide (PAP-3.2KD, sequence: VLSATDKTNVLAAW GKVGGNAPAFGAEALERM) is one of the main components of pilose antler. PAP-3.2KD may decrease the expression of TGF-β1 and further reduce SMADs, which are the target proteins of TGF-β1 signal transduction. Ultimately, the TGF-β/SMAD signaling pathway is inhibited, which thereby ameliorates histopathological damage, myocardial fibrosis, and apoptosis in adriamycin-induced myocardial tissues (61).

A total of 1,697 endogenous peptides in hypertrophic scar tissues and matched normal skin were identified using liquid chromatography-mass spectrometry/mass spectrometry. Among these, 78 peptides were highly expressed in normal tissues compared with the corresponding hypertrophic scar tissues. The peptides with fat solubility (aliphatic index > 100) and high stability (instability index < 0) were further screened. Considering that endogenous peptides were derived through hemoglobin reduction in hypertrophic scar tissues, LYENRL and ASGVAVSDGVIKV (ASGVA) were selected for further assessment of biological function. In addition, LYENPL could inhibit the proliferation of human skin fibroblast cells at a lower dose compared with ASGVA. LYENRL could reduce SMAD2/3 phosphorylation and the SMAD2/3/4 complexes to the SMAD Binding element, thereby inhibiting TGF-β1/SMAD signaling. Additionally, LYENRL can inhibit the proliferation, migration, and extracellular matrix production of scar fibroblasts in a concentration-dependent manner (62).

A region termed the SMAD4-binding domain (SBD) was identified, which was an amino-terminal region of transactivation 2 of p65 (a main subunit of NF-κB), associated with the mad homology 1 domain of SMAD4 to inhibit bone morphogenetic protein signaling. Based on the SBD, a cell-permeable SBD peptide (sequence: RRRRRRRGGGQAGEGTLSEALLHLQF) was designed and synthesized, which could block the association of p65 with SMAD4. In addition, the SBD peptide enhances BMP2-induced osteoblast differentiation and mineralization (63).

**Figure 10:**
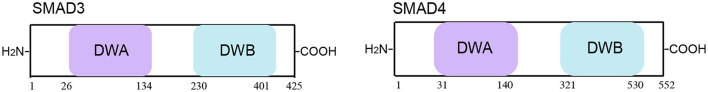
Schematic representation of the SMAD3/4 protein. SMAD3/4 includes 12 functional domains, with Domain A in dwarfin family proteins (DWA) and Domain B in dwarfin family proteins (DWB).

APNp could suppress mitochondrial and ATF4-CHOP apoptosis pathways in a SMAD3-dependent manner by targeting GSK-3β. Thus, neural survival was promoted following intracerebral hemorrhage injury in the diabetic setting (60) (Figure 10; Table 11).

**Table 11:** Peptide regulators targeting SMAD3/4.

**Name**	**Biological effect**	**Sequence**	**Function mechanism**
OA-GL17d	Activator	GLFKWHPRC GEEQSMWT	Acting on TGF-β1 through miR-663a to activate the SMAD signaling pathway
APNp	Activator	LQVYGDGDH NGLYADNVN	Suppressing mitochondrial and ATF4-CHOP apoptosis pathways in a SMAD3-dependent manner
PAP-3.2KD	Inhibitor	VLSATDKTNVLAAWGK VGGNAPAFGAEALERM	Decreasing the expression of TGF-β1 and further reducing SMADs
LYENRL	Inhibitor	LYENRL	Reducing SMAD2/3 phosphorylation and SMAD2/3/4 complex to SMAD Binding Element
SBD peptide	Inhibitor	RRRRRRRGGGQAG EGTLSEALLHLQF	Blocking the association of p65 with SMAD4

## 8. Conclusion and outlook

In summary, we have briefly explored the importance of the Wnt/β-catenin signaling pathway in human diseases. This signaling pathway is mainly involved in regulating biological processes such as cell proliferation, differentiation, and migration. In the Wnt/β-catenin signaling pathway, Wnt family ligands transduce signals to the Wnt/β-catenin signaling cascade through LRP5/6 and FZD receptors. Therefore, targeting molecules in the Wnt/β-catenin signaling pathway may modulate human diseases by activating or inhibiting this pathway, thereby affecting biological processes. Given recent research advancements, we summarize the peptide modulators in the Wnt/β-catenin signaling pathway. Studies have indicated that peptide regulators regulate the Wnt/β-catenin signaling pathway by targeting and antagonizing or agonizing molecules in the pathway.

Peptide regulators of the Wnt/β-catenin signaling pathway have shown promising results in preclinical studies. They have the potential to treat several diseases such as cancer, osteoporosis, and Alzheimer's disease. A recent study has shown that the interaction of the protein FAM83A with catenin is very important for the regulation of the development process of pancreatic cancer. Multiple blocking short peptide drug precursors targeting FAM83A catenin were screened and obtained. Their effectiveness was verified in animal models such as zebrafish and mice (131). In the future, peptide regulators could be developed into therapeutics that target specific diseases with great efficacy and fewer side effects than current treatments. Additionally, the development of peptide drug delivery systems could improve the bioavailability and pharmacokinetic properties of these agents. Furthermore, combining peptide regulators with other signaling pathway inhibitors could offer synergistic effects in treating diseases such as cancer. More research on the safety and efficacy of these peptide regulators is needed to fully understand their potential therapeutic benefits. However, polypeptide drugs also have some potential challenges, such as unstable physical and chemical properties, a short half-life, a fast clearance rate, and a lack of an effective drug delivery system, which are worthy of further exploration in future studies. Overall, peptide regulators of the Wnt/β-catenin signaling pathway have promising future perspectives as potential therapeutics for a range of diseases.

## Author contributions

NZ and HS: conceptualization. NZ, HS, BC, and HH: writing. CL: supervision. YC and WC: project administration. All authors have read and agreed to the published version of the manuscript.
